# HDAC1 and HDAC2 Double Knockout Triggers Cell Apoptosis in Advanced Thyroid Cancer

**DOI:** 10.3390/ijms20020454

**Published:** 2019-01-21

**Authors:** Ching-Ling Lin, Ming-Lin Tsai, Chun-Yu Lin, Kai-Wen Hsu, Wen-Shyang Hsieh, Wei-Ming Chi, Li-Chi Huang, Chia-Hwa Lee

**Affiliations:** 1 Department of Endocrinology and Metabolism, Cathay General Hospital, Taipei 10630, Taiwan; work5halfday@cgh.org.tw; 2 Department of Internal Medicine, School of Medicine, College of Medicine, Taipei Medical University, Taipei 11031, Taiwan; 3 Department of General Surgery, Cathay General Hospital, Taipei 10630, Taiwan; hyperexias@yahoo.com.tw; 4 Institute of Bioinformatics and Systems Biology, National Chiao Tung University, Hsinchu 30068, Taiwan; chunyulin.bi99g@g2.nctu.edu.tw; 5 Bioinformatics Center, Institute for Chemical Research, Kyoto University, Kyoto 611-0011, Japan; 6 Institute of New Drug Development, China Medical University, Taichung 40402, Taiwan; kwhsu@mail.cmu.edu.tw; 7 Research Center for Tumor Medical Science, China Medical University, Taichung 40402, Taiwan; 8 Department of Medical Laboratory, Taipei Medical University—Shuang Ho Hospital, New Taipei City 23561, Taiwan; 12638@s.tmu.edu.tw; 9 Department of Clinical Pathology, Taipei Medical University—Shuang Ho Hospital, New Taipei City 23561, Taiwan; rc4202@tmu.edu.tw; 10 School of Medical Laboratory Science and Biotechnology, College of Medical Science and Technology, Taipei Medical University, Taipei 11031, Taiwan; 11 Ph.D. Program in Medicine Biotechnology, College of Medicine, Taipei Medical University, Taipei 11031, Taiwan; 12 TMU Research Center of Cancer Translational Medicine, Taipei 11031, Taiwan

**Keywords:** anaplastic thyroid carcinoma, squamous thyroid carcinoma, histone deacetylase inhibitor, CRISPR/Cas9, knockout

## Abstract

Anaplastic thyroid carcinoma (ATC) and squamous thyroid carcinoma (STC) are both rare and advanced thyroid malignancies with a very poor prognosis and an average median survival time of 5 months and less than 20% of affected patients are alive 1 year after diagnosis. The clinical management of both ATC and STC is very similar because they are not particularly responsive to radiotherapy and chemotherapy. This inspired us to explore a novel and effective clinically approved therapy for ATC treatment. Histone deacetylase inhibitor (HDACi) drugs are recently FDA-approved drug for malignancies, especially for blood cell cancers. Therefore, we investigated whether an HDACi drug acts as an effective anticancer drug for advanced thyroid cancers. Cell viability analysis of panobinostat treatment demonstrated a significant IC50 of 0.075 µM on SW579 STC cells. In addition, panobinostat exposure activated histone acetylation and triggered cell death mainly through cell cycle arrest and apoptosis-related protein activation. Using CRISPR/Cas9 to knock out *HDAC1* and *HDAC2* genes in SW579 cells, we observed that the histone acetylation level and cell cycle arrest were enhanced without any impact on cell growth. Furthermore, *HDAC1* and *HDAC2* double knockout (KO) cells showed dramatic cell apoptosis activation compared to *HDAC1* and *HDAC2* individual KO cells. This suggests expressional and biofunctional compensation between HDAC1 and HDAC2 on SW579 cells. This study provides strong evidence that panobinostat can potentially be used in the clinic of advanced thyroid cancer patients.

## 1. Introduction

According to the National Cancer Institute, there are over 56,000 new cases of thyroid cancer in the USA each year and the majority of those diagnosed are papillary thyroid cancer, which is the most common type of thyroid cancer. Among all thyroid cancers, squamous thyroid carcinoma (STC) and anaplastic thyroid carcinoma (ATC) are rare neoplasms and have been reported in less than 1–2% of all thyroid cases [1,2]. STC and ATC are typically diagnosed as advanced thyroid malignancies with infiltration in adjacent organs, including the larynx, esophagus and trachea with a poor prognosis, where the median overall survival time is 6 months in clinical patients [3]. Due to the extremely aggressive behavior of ATC, the American Joint Committee on Cancer (AJCC) defines all of its stages as stage IV [4]. In the clinical management of advanced thyroid malignancy, surgical treatment is not always possible and the available radiotherapy/chemotherapy for thyroid cancer is always relatively ineffective [5]. Therefore, it is necessary to research highly efficient and low toxicity drugs to treat advanced thyroid cancers.

Histone modification is a covalent posttranslational modification (PTM) to histone proteins, which includes methylation, phosphorylation, acetylation, ubiquitylation and sumoylation. Among these PTMs, the regulation of histone acetylation and methylation have been well investigated in tumorigenesis, which manipulates the activation/inactivation of both oncogenic and tumor suppressive gene transcription. Histone deacetylases (HDACs) are epigenetic regulators that coordinate histone proteins, chromatin conformation and protein-DNA interactions. In many respects, histone deacetylase inhibitors (HDACis) represent prototypical epigenetic agents that act by modifying gene expression to restore the normal differentiation or death programs of transformed cells. To date, the USA FDA has approved four HDACis, including vorinostat (SAHA, 2006), romidepsin (FK228, 2009), belinostat (PXD101, 2014) and panobinostat (LBH-589, 2015), which are widely used as anticancer drugs, especially for refractory cutaneous and peripheral T cell lymphoma and multiple melanoma. Increasing evidence suggests that epigenetic alternations (DNA methylation, acetylation and chromatin modification) play key roles in thyroid cancer development, as well as cancer cell growth and differentiation [6,7,8,9,10]. Previous studies have shown that epigenetic modification causes gene silencing, which substantially decreases the responsiveness of thyroid tumors to radioiodine therapy [11,12,13]. Furthermore, epigenetic gene modifications also contribute to the dysregulation of both cell proliferation and cancer development, including apoptosis and cancer cell migration and invasion abilities.

In the current study, we investigated whether HDACi drugs, including panobinostat, belinostat, vorinostat and valproic acid, possess anticancer activity against advanced thyroid cancer, especially for SW579 STC cells. Further epigenetic alterations involved in cell cycle arrest and apoptosis were also investigated. Using our developed techniques, which combine lentiviral transfection and noninvasive apoptosis detection sensor (NIADS) analysis [14], we were able to quantitatively and kinetically analyze apoptotic cell death in HDACi-treated SW579 cells. HDAC1 and HDAC2 are two of the most investigated proteins in balancing histone acetylation and chromatin remodeling. By knocking out HDAC1 and HDAC2 using clustered regularly interspaced short palindromic repeats (CRISPR/Cas9), we investigated the impact of HDAC loss and how acetylation is regulated in thyroid cancer cells and observed the downstream signaling pathways and cell cycle regulation. In summary, this study could help us establish the basis of the pharmacological effects on HDAC regulation in future HDACi drug design targeting human advanced thyroid cancer.

## 2. Results

### 2.1. FDA-Approved HDACi Induced SW579 Cell Apoptosis

**Figure 1:**
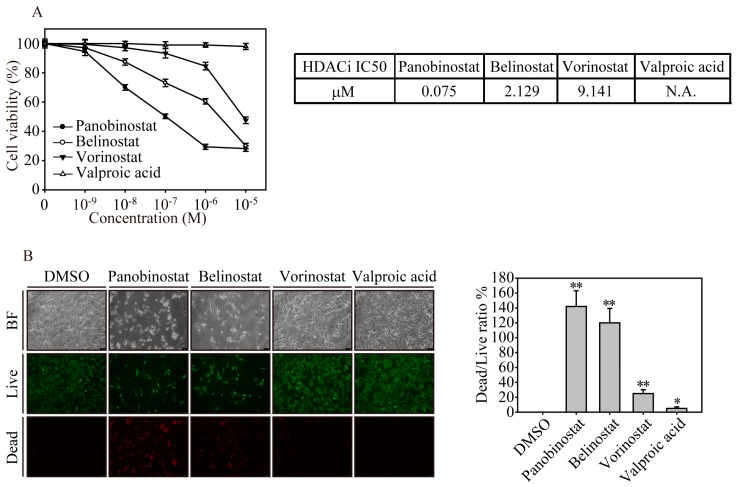
FDA-approved HDACi drugs significantly induced cell apoptosis in SW579 squamous-cell thyroid carcinoma (STC). (**A**) Cell viability of SW579 cells treated with four HDACi drugs at different concentrations (0.001, 0.01, 0.1, 1 and 10 µM) for 24 h analyzed by an MTT assay. The IC50 of HDACi drugs was the drug concentration that induced a 50% inhibition of cell viability. The cell viability values are presented as the means and standard deviation. The experiment was conducted at least in triplicate. (**B**) Live/dead cell viability assay. The brightfield and fluorescence images of HDACi-treated SW579 cells at 1 µM for 24 h. The cells were costained with 1 µM calcein-AM and 10 µM PI and live/dead cells were analyzed with fluorescence microscopy. The viable cells showed green fluorescence with light emission at a wavelength of 488 nm, whereas the dead cells showed red fluorescence in the nucleus with light emission at a wavelength of 532 nm. The ratio of live/dead cells after HDACi treatments was plotted with bars. Scale bar represents 10 µm, and the magnification is 100×. Data are presented as the mean and standard deviation. Data were analyzed with Student’s *t*-test and all *p*-values were two-sided. *p*-values less than 0.05 are indicated with an asterisk and *p*-values less than 0.01 are indicated with two asterisks.

Squamous-cell thyroid carcinoma (STC) cells were treated with different concentrations of four FDA-approved clinical HDACi drugs (panobinostat, belinostat, vorinostat and valproic acid) for the cell viability assay (Figure 1A). After HDACi drug exposure for 24 h, cells treated with panobinostat had a relatively low cell viability at 0.1 µM (50%) compared to belinostat (73.1%), vorinostat (93.3%) and valproic acid (99%) treatments. In addition, by investigating different doses of panobinostat treatment, SW579 cells had a lower cell viability at 1 and 10 µM (29.4% and 28.2%, respectively), whereas belinostat and vorinostat reached the maximum anticancer effect at 10 µM (30% and 47.5%, respectively) treatments. After analysis of the IC50, we observed that the IC50 of panobinostat is 0.075 µM, whereas belinostat and vorinostat have IC50 drug concentrations of 2.129 and 9.141 µM, respectively. Using a live/dead cell assay (Figure 1B), it is clear that exposure to 1 µM panobinostat and belinostat for 24 h caused significant cell death effects (red), with 142% and 120% dead/live cell ratios (*p* < 0.01), respectively, whereas vorinostat and valproic acid had relatively minor effects on cell death in SW579 cells. These cell viability results clearly indicate that panobinostat is one of the most effective anticancer drugs among the HDACi drugs on squamous-cell thyroid carcinoma of advanced thyroid cancer.

### 2.2. HDACi Induced Histone Acetylation and Apoptosis-Related Protein Expression

**Figure 2:**
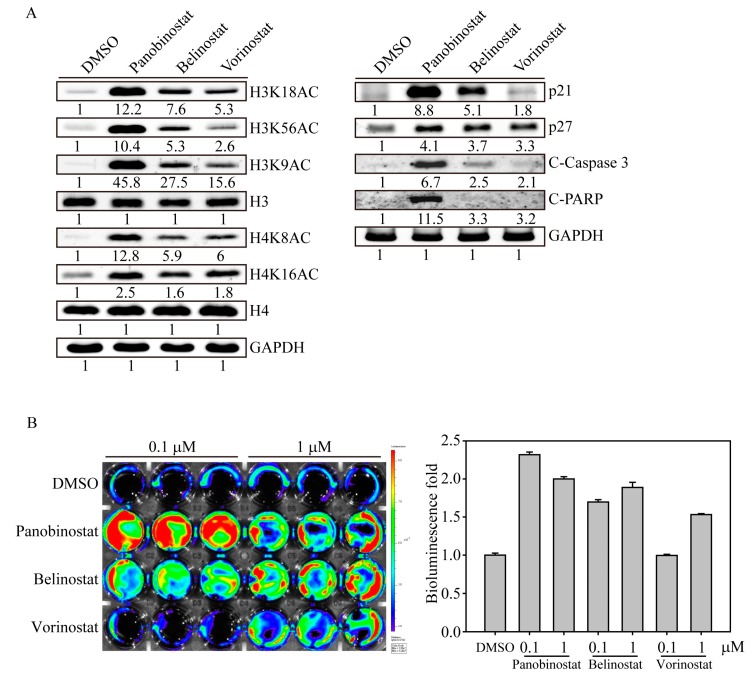
HDACi-induced histone acetylation and apoptosis-related protein expression. (**A**) HDACi drugs (panobinostat, belinostat and vorinostat) induced acetylation, cell cycle and apoptotic protein expression in SW579 cells. After 6 h of drug treatment, the cell lysate was collected and immunoblotted with different H3 (H3K9AC, H3K18AC and H3K56AC) and H4 (H4K8AC and H4K16AC) histone acetylation antibodies. After SW579 cells were treated for 24 h, the cell lysate was analyzed for cell cycle (p21 and p27) and apoptotic-related (C-caspase 3: cleaved caspase 3 and C-PARP: cleaved PARP) protein expression. Densitometric analysis of all western-blot was performed by ImageJ software. The protein expressions were normalized with their internal control such as H3, H4 or GAPDH expressions and shown as fold of control. (**B**) An IVIS image of HDACi-induced cell apoptosis in NIADS stably expressing SW579 (NIADS-KSW579) cells. The cells received an HDACi or DMSO in a dose-dependent manner (0.1 and 1 µM) for 24 h and the luciferase activity was analyzed (presented as the photon flux). Cells in a 24-well plate were exposed to luciferin at a concentration of 1.5 mg/mL and analyzed in an IVIS 200 Spectrum imaging system. The yellow and red colors indicate high photon counts of bioluminescence activity, whereas the green and blue colors present low photon counts of bioluminescence activity. The intense luciferase activity indicates apoptosis signals from NIADS.

Next, we investigated the acetylation sites of the histone complex that are activated by HDACi drugs in SW579 cells (Figure 2A). According to the cell viability results, we excluded valproic acid in the following investigations due to its minor effect on SW579 cells. After 6 h of HDACi treatment, panobinostat significantly induced histone H3 acetylation at amino acids 9, 18 and 56, whereas histone H4 was acetylated at sites 8 and 16. In contrast, belinostat was the second most effective drug that induced H3 and H4 histone acetylation in SW579 cells compared to vorinostat treatment. Furthermore, we also observed that panobinostat is the only drug that significantly induced p21 protein expression and activated cell apoptosis-related proteins, such as caspase and PARP (C-caspase and C-PARP), in SW579 cells, whereas GAPDH protein expression remained unchanged.

In a previous study, we established a bioluminescence-based live cell noninvasive apoptosis detection sensor (NIADS) system to evaluate the quantitative and kinetic characteristics of apoptotic cell death [14]. Using this assay, we were able to determine apoptotic events by simply measuring the bioluminescence activity in live cells. In this study, we used NIADS stably expressing SW579 cells (NIADS-SW579 cells), which were treated with different doses of HDACi drugs for 24 h and analyzed bioluminescence activity with in vivo imaging system (IVIS) (Figure 2B). The bioluminescence activity of NIADS-SW579 cells showed significant increases for the 0.1 and 1 µM panobinostat and belinostat treatments compared with the DMSO control. In addition, cells treated with 1 µM vorinostat had a higher bioluminescence activity than cells treated with 0.1 µM vorinostat or DMSO. However, we observed that the bioluminescence activity of cells treated with 1 µM panobinostat was less than that of cells treated with 0.1 µM panobinostat, which may be due to a strong apoptotic event causing less cell survival in 1 µM panobinostat-treated SW579 cells.

### 2.3. Panobinostat Dose-Dependently Induced Histone Acetylation and Apoptosis-Related Protein Expression in SW576 Cells

**Figure 3:**
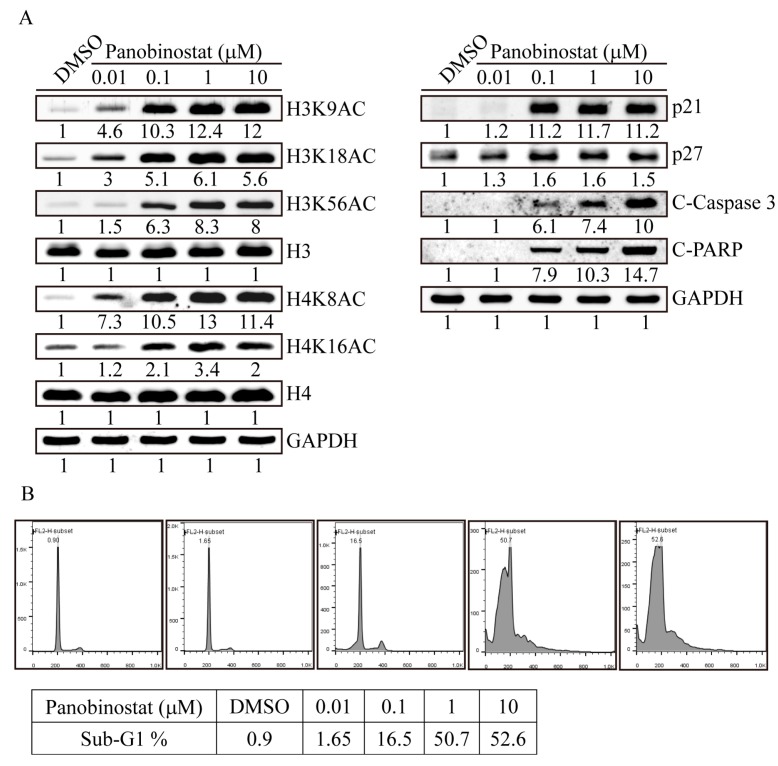
Panobinostat dose-dependently induced histone acetylation and apoptosis-related protein expression in SW579 cells. (**A**) Cell lysates from SW579 cells treated with different concentrations (0.01 to 10 µM) of panobinostat for 6 h were immunoblotted with different H3 (H3K9AC, H3K18AC and H3K56AC) and H4 (H4K8AC and H4K16AC) histone acetylation antibodies. Cell lysates of SW579 cells treated with panobinostat for 24 h were analyzed for cell cycle (p21 and p27) and apoptotic-related (C-caspase 3: cleaved caspase 3 and C-PARP: cleaved PARP) protein expression. The H3, H4 and GAPDH immunoblots served as internal controls. Densitometric analysis of all western-blot was performed by ImageJ software. The protein expressions were normalized with their internal control such as H3, H4 or GAPDH expressions and shown as fold of control. (**B**) Cell cycle analysis of dose-dependent panobinostat treatments from 0.01 to 10 μM on SW579 cells by flow cytometry. Apoptotic cells were determined by the sub-G1 cell population.

With various concentrations of panobinostat treatment on SW579 cells for 6 h, 0.01 µM panobinostat significantly induced histone H3K18 and H4K8 acetylation sites (Figure 3A), whereas H3K9, H3K56 and H4K16 were activated at 0.1 µM of panobinostat addition. Furthermore, similar to the observations described above, panobinostat dramatically induced p21 protein expression and activated apoptosis signals in cells treated with 0.1 µM for 24 h. Next, we used flow cytometry to confirm panobinostat-induced SW579 cell apoptosis (Figure 3B). After dose-dependent panobinostat treatments, SW579 cells had a significant sub-G1 apoptotic cell accumulation at a concentration of 0.1 µM with 16.5% of all cell populations, whereas 1 and 10 µM panobinostat treatments induced 50.7% and 52.6% sub-G1 apoptotic cells of all cell populations, respectively. These results indicated that panobinostat, as a pan-HDACi, efficiently induced apoptosis of STC cells at very low concentrations. Additionally, panobinostat-induced cell apoptosis may potentially occur through H3K9, H3K56 and H4K16 histone acetylation regulation in SW579 cells.

### 2.4. HDAC1 and HDAC2 Gene Editing through the CRISPR/CAS9 System

**Figure 4:**
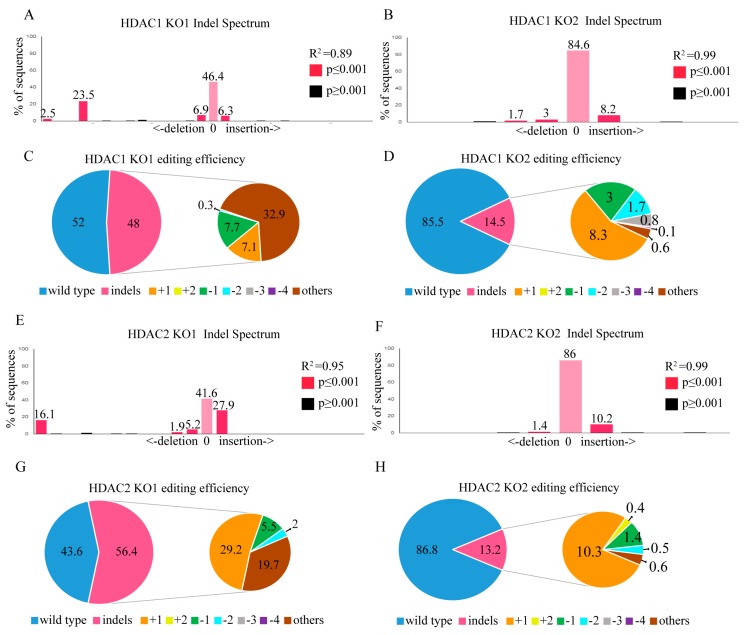
*HDAC1* and *HDAC2* gene editing of SW579 cells using the CRISPR/Cas9 system. Scrambled (SC) sgRNA and *HDAC1* sgRNA were delivered to SW579 cells by lentivirus. After transduction, DNA from virus-infected cells was purified and subjected to Sanger sequencing of *HDAC1* exon 2. The TIDE algorithm analysis is shown for (**A**) *HDAC1* KO1 and (**B**) *HDAC1* KO2 virus-transfected SW579 cells compared to SC SW579 cells. The pie charts show the percentages of indels in the *HDAC1* gene edited by *HDAC1* (**C**) KO1 and (**D**) KO2 lentivirus. The gene editing efficiency of the two KO cells is shown in pink, while the two most common other mutations and +1 are shown in brown and yellow colors, respectively. SC sgRNA and *HDAC2* sgRNA were delivered to SW579 cells by lentivirus. After transduction, DNA from virus-infected cells was purified and subjected to Sanger sequencing of *HDAC2* exon 2. The TIDE algorithm analysis is shown for (**E**) *HDAC2* KO1 and (**F**) *HDAC2* KO2 virus-transfected SW579 cells compared to SC SW579 cells. The pie charts show the percentages of indels in the *HDAC2* gene edited by *HDAC2* (**G**) KO1 and (**H**) KO2 lentivirus. The gene editing efficiency of the two KO cells is shown in pink, while the two most common other mutations and +1 are shown in brown and yellow colors, respectively.

Next, we investigated the utility of CRISPR/Cas9 genome editing by targeting two custom-designed protospacers on *HDAC1* (NM_004964.2) on chromosome 1 and the *HDAC2* (NM_001527.3) locus on chromosome 6 with a lentiviral delivery system using the MIT CRISPR design website (http://crispr.mit.edu). SW579 cells transfected with scrambled (SC) lentivirus produced a wild-type *HDAC1* sequence (Supplementary Figure S1A,B), indicating that no gene editing occurred. In contrast, SW579 cells transfected with *HDAC1* KO1 lentivirus carrying protospacer 1 (Supplementary Figure S1C) had more significant multiple gene disruptions at the predicted cleavage sites (red arrowhead) than *HDAC1* KO2 lentivirus-transfected cells (Supplementary Figure S1D). Furthermore, TIDE analysis demonstrated that *HDAC1* KO1 cells (Figure 4A) had a higher gene editing efficiency than *HDAC1* KO2 cells (Figure 4B), with 48% and 14.5% of the cell pool edited, respectively. The most frequent mutation in the *HDAC1* KO1 cell pool was other mutations (85.2%, Figure 4C), whereas the frequently predicted mutation in the *HDAC1* KO2 cell pool was a 1-bp insertion (8.3%, Figure 4D). Compared to *HDAC1* KO2 cells, SW579 cells transduced with *HDAC1* KO1 caused more significant gene disruptions in the targeted regions, with mutations primarily at the predicted cleavage sites (Supplementary Figure S1E,F). However, both protospacer 1- and protospacer 2-containing HDAC2 lentivirus targeted the plus strand of exon 1 on the *HDAC2* gene. Sanger sequencing showed no evidence of gene editing on SC lentivirus-transduced SW579 cells (Supplementary Figure S1G,H). Compared to *HDAC2* KO2 cells (Supplementary Figure S1J), *HDAC2* KO1 cells (Supplementary Figure S1I) showed significant multiple gene disruptions at the predicted cleavage sites (red arrowhead). Using TIDE analysis, *HDAC2* KO1 cells (Figure 4E) also showed more considerable gene editing efficiency than *HDAC2* KO2 cells (Figure 4F), with 56.4% and 10.3% of the cell pool edited, respectively. The most frequent mutation in the *HDAC2* KO1 cell pool was a 1-bp insertion (29.2%, Figure 4G), whereas the frequently predicted mutation in the *HDAC2* KO2 cell pool was a 1-bp insertion (10.3%, Figure 4H). In addition, only *HDAC2* KO1 caused significant gene disruptions in the targeted regions, whereas no gene disruptions were observed in *HDAC2* KO2 SW579 cells, with mutations primarily at the predicted cleavage sites (Supplementary Figure S1K,L).

### 2.5. HDAC1 and HDAC2 Knockout Activates H3 and H4 Histone Acetylations

**Figure 5:**
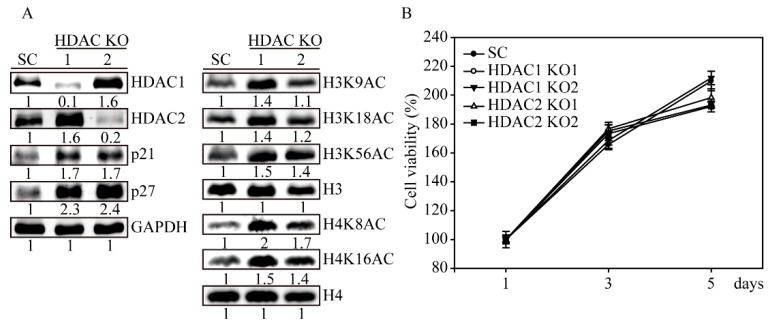
*HDAC1* and *HDAC2* KO SW579 cells induced H3 and H4 acetylation and cell arrest proteins. (**A**) Immunoblot analysis of gene expression in *HDAC1* and *HDAC2* KO SW579 cells. The cell lysates from SC, *HDAC1* KO1, *HDAC1* KO2, *HDAC2* KO1 and *HDAC2* KO2 cells were immunoblotted with different H3 (H3K9AC, H3K18AC and H3K56AC) and H4 (H4K8AC and H4K16AC) histone acetylation antibodies. SW579 cell lysates were also examined for cell cycle (p21 and p27) protein expression. The H3, H4 and GAPDH immunoblots served as internal controls. Densitometric analysis of all western-blot was performed by ImageJ software. The protein expressions were normalized with their internal control such as H3, H4 or GAPDH expressions and shown as fold of control. (**B**) MTT cell viability assay of SC, *HADC1* and *HDAC2* KO cells for 5 days. The cell viability values are presented as the means and standard deviation. The experiment was conducted at least in triplicate.

Next, we evaluated HDAC1 and HDAC2 protein expression in the gene-edited SW579 cells by western blotting (Figure 5A). Similar to the abovementioned genomic results, the protein expression levels of both *HDAC1* and *HDAC2* virus-transfected KO1 SW579 cells were significantly decreased compared to SC virus-transfected cells. In addition, *HDAC1* KO1 cells slightly gained higher HDAC2 protein expression levels, whereas *HDAC2* KO1 cells gained higher HDAC1 protein expression levels in both gene-edited SW579 cells. This observation proves evidence that HDAC1 and HDAC2 have complementary effects. In the screening of histone acetylation in SW579 cells, we observed that *HDAC1* KO1 cells obtained higher H3K9, H3K18, H3K56, H4K8 and H4K16 acetylation sites than *HDAC2* KO1 cells. The cell arrest proteins p21 and p27 were also dramatically induced in *HDAC1* cells. Next, we examined this controversial result by an MTT cell viability assay (Figure 5B). During a five-day observation, SC, *HDAC1* KO1, *HDAC1* KO2, *HDAC2* KO1 and *HDAC2* KO2 cells showed no differences in cell proliferation, confirming that the lack of HDAC1 or HDAC2 may compensate for each other by protein expression or cell signaling crosstalk.

### 2.6. HDAC1 and HDAC2 Double Knockout Induced Cell Apoptosis in SW579 Cells

**Figure 6:**
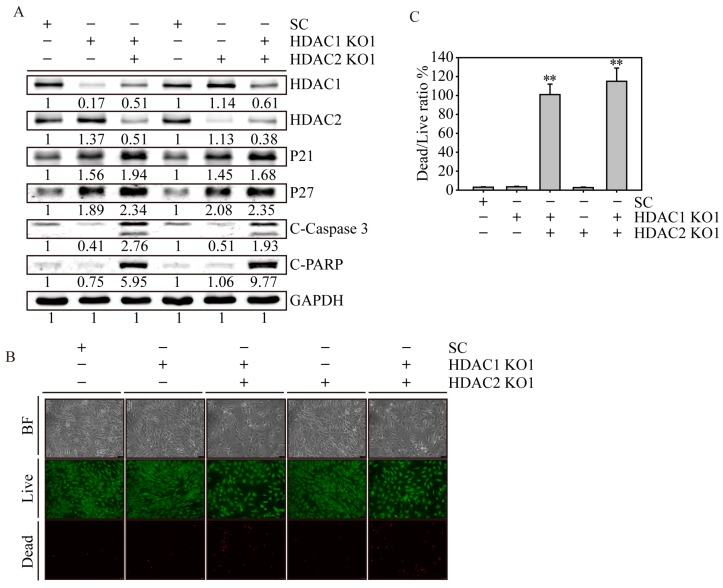
*HDAC1* and *HDAC2* double knockout SW579 cells induced cell cycle arrest and apoptosis. (**A**) Immunoblot analysis of protein expression in SC, *HDAC1*, *HDAC2* and *HDAC1*,*2* KO SW579 cells. The cell lysate was analyzed for HDAC1 and HDAC2 expression and cell cycle (p21 and p27) and apoptosis-related (C-Caspase 3: cleaved caspase 3 and C-PARP: cleaved PARP) protein expression. GAPDH immunoblots served as internal controls. Densitometric analysis of all western-blot was performed by ImageJ software. The protein expressions were normalized with their internal control such as H3, H4 or GAPDH expressions and shown as fold of control. (**B**) Live/dead cell viability assay of SC, *HDAC1*, *HDAC2* and *HDAC1*,*2* KO SW579 cells. The cells were costained with 1 µM calcein-AM and 10 µM PI and live/dead cells were analyzed using fluorescence microscopy. The viable cells showed green fluorescence with light emission at a wavelength of 488 nm, whereas the dead cells showed red fluorescence in the nucleus with light emission at a wavelength of 532 nm. The brightfield images show the morphology of gene-edited SW579 cells. Scale bar represents 10 µm, and the magnification is 100×. (**C**) The dead/live cell ratio of gene-edited SW579 cells. Data are presented as the mean and standard deviation. Data were analyzed with Student’s *t*-test and all *p*-values were two-sided. *p*-values less than 0.01 are indicated with two asterisks.

To investigate the compensation effect of HDAC1 and HDAC2 in maintaining cell survival on SW579 cells, we used either *HDAC1* or *HDAC2* KO cells that were additionally transfected with *HDAC2*- and *HDAC1*-targeting lentivirus to create two individual *HDAC1* and *HDAC2* double knockout cells (HDAC1,2 KO). Using western blotting (Figure 6A), we observed that the HDAC1 protein expression level was significantly decreased in *HDAC1* KO and *HDAC1*,*2* KO cells, whereas the HDAC2 protein expression level was significantly decreased in *HDAC2* KO and *HDAC1*,*2* KO cells compared to SC SW579 cells. Interestingly, p21 and p27 cell cycle arrest-related protein expression levels were dramatically increased in *HDAC1*, *HDAC2* and *HDAC1*,*2* KO cells. Notably, apoptosis-related proteins, such as cleaved caspase 3 and PARP, were only observed in *HDAC1*,*2* KO cells compared to SC, *HDAC1* and *HDAC2* cells. Using a live/dead cell assay (Figure 6B), we confirmed that two individual *HDAC1*,*2* KO cells showed substantial apoptotic events (red), with 101% and 115% dead/live cell ratios (*p* < 0.01), whereas the cell death of *HDAC1* and *HDAC2* KO cells was relatively minorly affected. This result showed strong evidence that maintaining both HDAC1 and HDAC2 is essential for cell survival in SW579 cells. Therefore, using pan-HDACi drugs for advanced thyroid malignancy would be an effective clinical anticancer strategy in the future.

## 3. Discussion

During the last two decades, thyroid cancer has increased at a higher rate than any other cancer worldwide [15], which is attributed to the genetic and environmental changes that create epigenetic modifications. Most previous studies have focused on well-differentiated thyroid cancer (WDTC), including papillary thyroid cancer (PTC) and follicular thyroid cancer (FTC), because of their prevalence and the availability of samples [16]. According to the results, the epigenetic mechanisms of histone modification and miRNA regulation seem to play an important role in thyroid tumorigenesis [17,18,19]. In addition, global methylation of 83 primary WDTC tumors as well as 8 samples of adjacent normal tissues identified that DNA methylation is an important mechanism that regulates signaling pathways during WDTC development, whereas etoposide-induced 2.4 (*EI24*) and Wilms tumor 1 (*WT1*) are novel prognostic markers related to recurrence-free survival [20]. Based on the evidence presented in this study, considerable epigenetic alternations, such as HDAC1 and HDAC2, in advanced thyroid cancer were investigated and more epigenetic alterations are being studied.

By increasing histone protein acetylation, HDACis lead to DNA remodeling and transcriptional activation of genes. Panobinostat is an oral pan-HDAC inhibitor that has been approved for third-line therapy of relapsed multiple myeloma [21]. The study showed that ATC cell lines, including BHT-101, CAL-62 and 8305C, exposed to panobinostat showed loss of cell viability, inhibition of colony formation, arrest of the cell cycle and induction of apoptosis [22]. Furthermore, animal data confirmed the cytotoxic properties of panobinostat, showing that the drug significantly reduced the expression of the Ki67 proliferation marker and therefore impaired tumor growth of ATC xenografts. In this study, the mechanisms underlying the cytotoxic effect of panobinostat on the SW579 squamous-cell thyroid carcinoma cell line included both apoptosis induction and cell cycle arrest. Apoptosis was demonstrated by the increased percentage of cells observed in the live/dead assay, sub-G_1_, induction of NIADS and by the activation of both PARP and caspase 3. The effect of panobinostat on cell cycle progression showed a distinctive feature; the arrest in G_2_/M was observed after treatment with higher doses of the drug (approximately 0.1 µM). This observation is consistent with a previous study showing that HDACis typically determine cytotoxicity at higher doses, whereas they induce G_2_/M arrest at lower doses [23].

The quantification of apoptosis-related cell death is an integral component of exploring cell biology and responses to cellular stress and performing high-throughput drug screens [24]. However, currently available quantitative apoptosis assays, such as the annexin V/FITC assay, require extensive sample handling and substantial labor for cell harvest and analysis. Therefore, a quantitative apoptosis assay with easy handling (the NIADS assay) was established in our previous study [14], with notable advantages, such as relatively low cell numbers, trace amounts of bioluminescence, viral infection and quick assay. These unique strengths make apoptosis detection assays suitable for high-throughput cell-based screening of drug libraries and related applications. The limitation of this assay was that higher doses of panobinostat treatment on SW579 cells resulted in lower bioluminescence signals (Figure 2B). The disadvantage was due to the strong anticancer effect of panobinostat causing less SW579 cell survival on the plate, whereas NIADS detection requires cell membranes to remain intact. To improve this assay, we aim to integrate a reporter in the current NIADS system. Therefore, the bioluminescence signals of NIADS could be normalized with internal control reporter signals. This adjustment could provide more accurate and reliable apoptosis analysis in vitro and in vivo.

A previous study demonstrated that panobinostat inhibited cell viability and colony formation abilities, whereas enhanced cell cycle arrest and apoptosis induction on three ATC cell lines (BHT-101, CAL-62 and 8305C) [22]. In animal study, a SCID xenograft model showed intraperitoneal injection of panobinostat 20 mg/kg/day of panobinostat group significantly decreased tumor growth for 2.5-fold, compared to control mice. The recent study also confirms this finding by using belinostat and panobinostat on ATC and unclassified thyroid cancer cells [25], whereas animal model showed a prominent inhibition of BHP2-7 tumor growth of the tumors occurred in the belinostat intraperitoneal injected mice (100 mg/kg/day). To summarize, the current study once again confirms the anti-thyroid cancer ability by panobinostat or belinostat treatments, appears to be a promising therapeutic agent for the advanced cancer which is known not to respond to conventional therapy.

Previous studies have shown that knockdown of either *HDAC1* or *HDAC2* resulted in the sensitization of chronic lymphocytic leukemia cells to Trail-induced apoptosis [26] and a reduction in the proliferation of colon cancer cells [27]. These results indicate that the similar effects of the inactivation of either HDAC1 or HDAC2 lead to cancer cell growth suppression. In this study, we observed a compensatory mechanism for both HDAC1 and HDAC2 in SW579 cells. CRISPR/Cas9 mediated HDAC1 knockout, leading to HDAC2 protein upregulation, while HDAC2 ablation resulted in increased HDAC1 expression levels (Figure 5A). These results are consistent with other reported mouse knockout studies [28,29]. Thus, using pan-HDACi drugs to target multiple HDACs could be an effective antitumor strategy for clinical treatment of thyroid cancer and could potentially increase the survival rate of advanced thyroid cancer patients.

## 4. Materials and Methods

### 4.1. Cell Culture

The human gland epithelial squamous cell carcinoma cell line SW579 was purchased from the Bioresource Collection and Research Center (BCRC, Hsinchu, Taiwan). The cells were maintained in Dulbecco’s modified Eagle’s medium nutrient mixture F-12 (DMEM/F-12) (Gibco, Carlsbad, CA, USA). The cells were cultured with 10% (*v*/*v*) fetal bovine serum (FBS, Biological Industries, Kibbutz Beit Kaemek, Israel), 100 units/mL penicillin and 100 mg/mL streptomycin and were incubated at 37 °C with 5.0% CO_2_. The medium was replaced every two days and when cells reached 80% confluence, they were passaged using 0.25% trypsin/EDTA (Gibco, CA, USA).

### 4.2. MTT Cell Viability Assay

Cell viability was analyzed using a 3-(4,5-dimethylthiazol-2-yl)-2,5-diphenyltetrazolium (MTT) assay, which is based on the reduction of yellow MTT to purple formazan by living cells [30,31]. A total of 5 × 10^4^ SW579 cells were seeded in a 96 multiwell plate overnight before HDACi treatment. After 24 h, the medium was changed to fresh medium containing 1 g/mL MTT reagent for two h. DMSO was added as a solvent in each well and the absorbance OD at a wavelength of 570 nm was obtained.

### 4.3. Live/Dead Cell Assay

SW579 cells were seeded in a 12 multiwell plate at an appropriate cell density and cultured overnight. After the indicated drug treatment, the medium was replaced with culture medium containing 1 µM calcein-AM and 10 µM propidium iodide (PI) for 30 min of incubation. The cells were analyzed by the live/dead cell assay using fluorescence microscopy. The viable cells showed green fluorescence with light emission at a wavelength of 488 nm and the dead cells showed red fluorescence in the nucleus with light emission at a wavelength of 532 nm.

### 4.4. Cellular Bioluminescence (IVIS) Assay

Bioluminescence imaging was performed using an in vivo imaging system (IVIS; Xenogen) with a highly sensitive and cooled CCD camera mounted in a light-tight specimen box. SW579 cells were seeded in a 24-well multiwell plate and treated with the HDACi at concentrations of 0.1 and 1 µM for 24 h. d-Luciferin at a final concentration of 1.5 mg/mL was added to multiwell plates during imaging. The bioluminescent light from the cells was detected by the IVIS camera system and integrated, digitized and displayed. The light from each well was quantified and expressed as the total photon count using Living Image^®^ software 4.0 (Caliper, Alameda, CA, USA).

### 4.5. Flow Cytometry Analysis

SW579 cells (1 × 10^6^ cells/dish) were plated in a 6-cm dish and exposed to the HDACi for 24 h. SW579 cells were collected, washed once with PBS, fixed with 75% alcohol and analyzed with sub-G1 cell populations by flow cytometry (FACSCalibur, BD Biosciences, San Jose, CA, USA).

### 4.6. Protein Extraction, Western Blotting and Antibodies

For western blot analysis, SW579 cells were collected and washed once with ice-cold PBS, followed by the addition of radio-immunoprecipitation assay (RIPA) lysis buffer, which contained protease inhibitors. Fifty micrograms of protein from each sample was resolved by sodium dodecyl sulfate-polyacrylamide gel electrophoresis (SDS-PAGE) and transferred to a nitrocellulose membrane. The information of primary antibodies and the secondary antibodies used in this study is provided in Supplementary Table 1. All primary antibodies were used at a 1:1000 dilution with overnight hybridization, followed by a one-h incubation with a 1:4000 dilution of the secondary antibodies. Densitometric analysis of all western-blot was performed by ImageJ software (U.S. National Institutes of Health, Bethesda, MD, USA), whereas the protein expressions were normalized with its internal control such as H3, H4 or GAPDH expressions and shown as fold of control.

### 4.7. Lentivirus Production and Cell Transduction

HDAC1- and HDAC2-targeting lentiviral particles and NIADS lentivirus were produced by transient transfection of Phoenix-ECO cells (CRL-3214) using TransIT^®^-LT1 Reagent (Mirus Bio LLC, Madison, WI, USA). Guide oligonucleotides were phosphorylated, annealed and cloned into the BsmBI site of the lentiCRISPR v2 vector (Addgene, 52961, kindly provided by Feng Zhang) according to the Zhang laboratory protocol [32] (F. Zhang Laboratory, MIT, Cambridge, MA, USA). All plasmid constructs were verified by sequencing. The *HDAC1*, *HDAC2* and NIADS plasmids were cotransfected with pMD2.G (Addgene plasmid #12259) and psPAX2 (Addgene plasmid #12260), which were both kindly provided by Didier Trono, EPFL, Lausanne, Switzerland. Lentiviral particles were collected at 36 and 72 h and then concentrated with a Lenti-X Concentrator^®^ (Clontech, Mountain View, CA, USA). The lentiviral particles were analyzed by Q-PCR [31]. A total of 1 × 10^6^ SW579 cells were plated in a 6-cm dish and treated with lentivirus at an MOI of 5. Two days after transfection, the medium was replaced with medium containing 2.5 mg/mL puromycin for two days. Lentivirus-transfected cells were recovered two days before the experiments.

### 4.8. Sequencing of Single Guide RNA (sgRNA) Target Sites

Genomic DNA was extracted and PCR was used to amplify the *HDAC1* and *HDAC2* gene region using exon 2 DNA primers, which are listed in Supplementary Table 2, whereas sgRNA sequence were underlined. The PCR products were purified using a PCR clean-up purification kit and were sequenced by the Sanger method using forward PCR primers. The editing efficiency of the sgRNAs and the potential induced mutations were assessed using Tracking of Indels by Decomposition (TIDE) software (https://tide-calculator.nki.nl; Netherlands Cancer Institute, Amsterdam, The Netherlands), which only required two Sanger sequencing runs from wild-type and mutated cells.

### 4.9. Statistical Methods

All data are presented as the mean ± SD. Student’s *t*-test analysis was performed for the pairwise samples. All bar graphs were plotted using SigmaPlot graphing software (version 10.0, Systat software Inc, Chicago, IL, USA). The statistical analyses were performed using Statistical Package for the Social Sciences v.13 software (SPSS, Chicago, IL, USA). All statistical tests were two-sided. A *p*-value of 0.05 or less was considered to indicate statistical significance. *p*-values less than 0.05 are indicated with an asterisk and *p*-values less than 0.01 are indicated with two asterisks.

## 5. Conclusions

The 5-year survival rate of thyroid cancer is greater than 90%. However, the most aggressive and malignant thyroid cancer types, such as anaplastic thyroid carcinoma (ATC) and squamous thyroid carcinoma (STC), both have a very poor prognosis. In the current study, we observed that HDAC1 and HDAC2 have compensatory effects, which play important roles in maintaining cell survival in aggressive STC SW579 cells. By introducing sgRNAs of both *HDAC1* and *HDAC2* to edit these genes, cell cycle arrest and apoptotic events of SW579 cells were significantly enhanced. In addition, we observed that pan-HDACi drug treatment using panobinostat resulted in a substantially greater anticancer effect on SW579 cells compared to other HDACi drugs. This could also be effective in the clinical treatment of aggressive thyroid cancer.

## Supplementary Materials

Supplementary materials can be found at http://www.mdpi.com/1422-0067/20/2/454/s1.

## Abbreviations

STC	Squamous thyroid carcinoma
HDACi	Histone deacetylase inhibitor
NIADS	Noninvasive apoptosis detection sensor
KO	Knockout
CRISPR/Cas9	Clustered regularly interspaced short palindromic repeat
IVIS	in vivo imaging system
