# Supplementary material for: HDAC1 and HDAC2 Double Knockout Triggers Cell Apoptosis in Advanced Thyroid Cancer
Source: Int J Mol Sci. 2019 Jan 21;20(2):454. doi: 10.3390/ijms20020454 (PMC6359659; doi:10.3390/ijms20020454)
Supplement: Supplementary file 1 [file ijms-20-00454-s001.pdf]

Supplementary figure 1

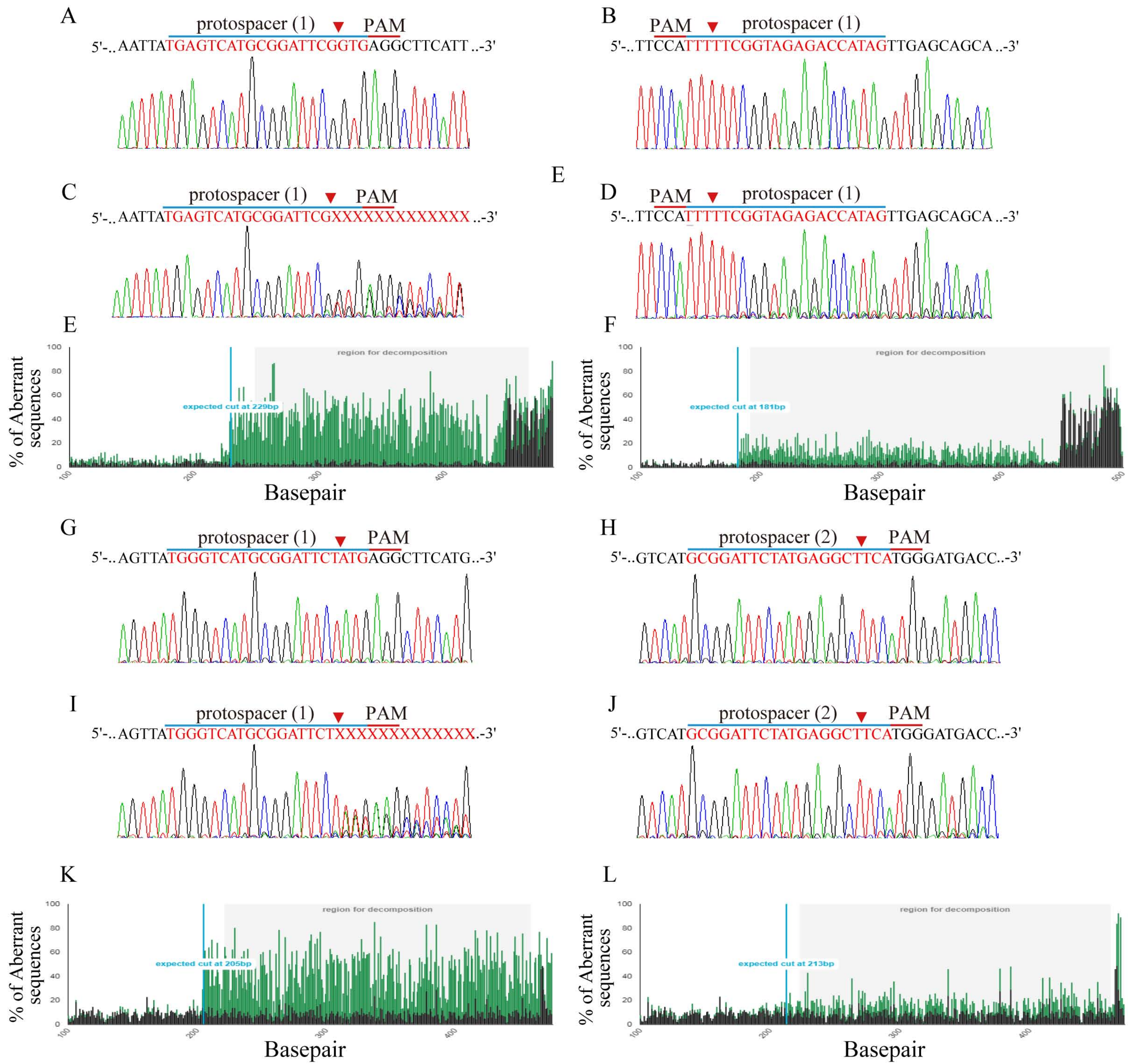

Supplementary table 1

Primary Antibody

Antibody	Catalog Number	Company Name (Origin)
GAPDH	sc-32233	Santa Cruz Biotechnology (Santa Cruz, CA, USA)
H3	GTX122148	GeneTex Inc. (Irvine, CA, USA)
H3K56AC	GTX60902	GeneTex Inc. (Irvine, CA, USA)
H3K18AC	GTX128943	GeneTex Inc. (Irvine, CA, USA)
H3K9AC	GTX630554	GeneTex Inc. (Irvine, CA, USA)
H4	GTX129560	GeneTex Inc. (Irvine, CA, USA)
H4K8AC	GTX633420	GeneTex Inc. (Irvine, CA, USA)
H4K16AC	GTX632067	GeneTex Inc. (Irvine, CA, USA)
p21	GTX629543	GeneTex Inc. (Irvine, CA, USA)
p27	GTX100446	GeneTex Inc. (Irvine, CA, USA)
C-PARP	#9541	Cell Signaling Technology (Danvers, MA, USA)
C-CASPASE-3	#9664	Cell Signaling Technology (Danvers, MA, USA)
p-AKT	#4060	Cell Signaling Technology (Danvers, MA, USA)
p-ERK	sc-7383	Santa Cruz Biotechnology (Santa Cruz, CA, USA)
HDAC1	GTX100513	GeneTex Inc. (Irvine, CA, USA)
HDAC2	GTX10964	GeneTex Inc. (Irvine, CA, USA)

Secondary Antibody

goat Anti-Rabbit IgG-HRP	sc-2004	Santa Cruz Biotechnology (Santa Cruz, CA, USA)
goat Anti-Mouse IgG-HRP	sc-2005	Santa Cruz Biotechnology (Santa Cruz, CA, USA)

Supplementary table 2

Primer sequence

Primer	Sequence	Assay
**HDAC1 exon2-1 sense**	CAC CGT GAG TCA TGC GGA TTC GGT G	Gene editing assay
**HDAC1 exon2-1 antisense**	AAA CCA CCG AAT CCG CAT GAC TCA C	Gene editing assay
**HDAC1 exon2-2 sense**	CAC CGC TAT GGT CTC TAC CGA AAA A	Gene editing assay
**HDAC1 exon2-2 antisense**	AAA CTT TTT CGG TAG AGA CCA TAG C	Gene editing assay
**HDAC2 exon2-1 sense**	CAC CGT GGG TCA TGC GGA TTC TAT G	Gene editing assay
**HDAC2 exon2-1 antisense**	AAA CCA TAG AAT CCG CAT GAC CCA C	Gene editing assay
**HDAC2 exon2-2 sense**	CAC CGG CGG ATT CTA TGA GGC TTC A	Gene editing assay
**HDAC2 exon2-2 antisense**	AAA CTG AAG CCT CAT AGA ATC CGC C	Gene editing assay
**HDAC1 DNA sense**	AGT GAG CTA GAC TGA ACC TTA	TIDE assay
**HDAC1 DNA antisense**	TGC TGT GAT TAC AGG CG	TIDE assay
**HDAC2 DNA sense**	CTC ATT CTT ATT CAT CTC CTA	TIDE assay
**HDAC2 DNA antisense**	AAT ATA ATG TGT AAT AAT GGA GTC	TIDE assay
**GUS realtime sense**	CCT GTT TAC TTG AGC AAG ACT GAT A	Q-PCR assay
**GUS realtime antisense**	CCT TTA GTG TTC CCT GCT AGA ATA	Q-PCR assay
